# Possible use of *ail *and *foxA *polymorphisms for detecting pathogenic *Yersinia enterocolitica*

**DOI:** 10.1186/1471-2180-10-211

**Published:** 2010-08-07

**Authors:** Ying Huang, Xin Wang, Zhigang Cui, Yuhuan Yang, Yuchun Xiao, Liuying Tang, Biao Kan, Jianguo Xu, Huaiqi Jing

**Affiliations:** 1 National Institute for Communicable Disease Control and Prevention, Chinese Center for Disease Control and Prevention, State Key Laboratory for Infectious Disease Prevention and Control, 102206, Beijing, China; 2 Fujian Provincial Centre for Disease Control and Prevention, 350001, Fuzhou, China; 3 National Institute for Viral Disease Control and Prevention, Chinese Center for Disease Control and Prevention, 100052, Beijing, China

## Abstract

**Background:**

*Yersinia enterocolitica *is an enteric pathogen that invades the intestinal mucosa and proliferates within the lymphoid follicles (Peyer's patches). The attachment invasion locus (*ail*) mediates invasion by *Y. enterocolitica *and confers an invasive phenotype upon non-invasive *E. coli*; *ail *is the primary virulence factor of *Y. enterocolitica*. The ferrioxamine receptor (*foxA*) located on the *Y. enterocolitica *chromosome, together with its transport protein, transports a siderophore specific for ferric ion. Currently, *ail *is the primary target gene for nucleic acid detection of pathogenic *Y. enterocolitica*.

**Results:**

The genes *ail *and *foxA *in 271 pathogenic and 27 non-pathogenic *Y. enterocolitica *strains isolated from China and 10 reference strains were sequenced, aligned, compared to the *ail *and *foxA *sequences of *Yersinia enterocolitica subsp. enterocolitica *8081 (Genbank: NC_008800), and analyzed for sequence polymorphism. The *ail *from the 282 strains showed 3 sequence patterns: 277 strains of serotypes O:3, O:9 and O:5, 27 with identical nucleic acid sequences formed pattern A1; 4 strains of serotype 1B/O:8 with identical nucleic acid sequences formed pattern A2; and one Chinese isolate 2/O:9 formed pattern A3. In the primary coding region of the *foxA *ORF (Genebank: X60447 nt 433-1866; nt 28 to 1,461 in the ORF), the sequences formed 3 groups and were further divided into 8 sequence patterns.

**Conclusion:**

The *ail *and *foxA *loci of pathogenic *Y. enterocolitica *have been analyzed. The *ail *sequence was highly conserved among the same serotype strains from different sources; and *foxA *was highly conserved among the pathogenic strains, although there was some sequence diversity. Fewer strains were used from outside China, which is a limitation of the study.

## Background

*Yersinia enterocolitica *is a food-borne pathogen [1] that causes a broad spectrum of clinical syndromes. A primary characteristic of the microorganism is that it penetrates the intestinal epithelial cells and replicates in lymphatic nodules, causing a wide variety of clinical and immunological manifestations [2-4]. All pathogenic *Y. enterocolitica *strains harbor *ail*, which is different from the *inv *sequence (which encodes a protein of similar function), and renders *Y. enterocolitica *capable of invading the intestinal epithelium. In addition, the Ail protein confers a serum resistance phenotype on *Y. enterocolitica *[5]. In contrast to *inv*, which exists in non-pathogenic as well as pathogenic strains of *Y. enterocolitica*, *ail *only exists in *Y. enterocolitica *strains epidemiologically related to human disease [6], and is therefore an important virulence marker. Environmental isolates not associated with disease have a non-functional *inv *and no *ail *[7].

Ferric ion uptake is essential for bacterial growth and survival. The supply of iron and production of the siderophore transport system is a central factor in infections with *Yesinia pestis *and *Y. enterocolitica*. Pathogenic *Y. enterocolitica *can be divided into 2 groups, those producing the siderophore, such as biotype 1B/O:8, and those producing no siderophore, as in serotypes O:3 and O:9 [8]; the latter take up ferric ion using ectogenic siderophores, such as ferrioxamin B and ferrioxamin E [9]. The 2 groups have different ferric ion uptake abilities, which may explain the differences in virulence among serotypes [10]. A 77 kDa receptor on the *Y. enterocolitica *outer membrane [11] combines with ferrioxamin to take up ferric ion rapidly [12]. This process is energy-dependent and requires the action of the TonB protein, part of a complex known as the Ton system. This complex undergoes a conformational change driven by the proton motive force (PMF), which interacts with the outer membrane receptors and activates transport [13]. The FoxA receptor of *Y. enterocolitica*, the ferrochrome receptor and the TonB-dependent receptor share high amino acid homology [14,15]. The *foxA *was chosen for study because it exists in all *Y. enterocolitica *strains.

Using polymorphic gene analysis, we show that combined detection of *ail *and *foxA *confirms the identity of pathogenic *Y. enterocolitica*.

## Methods

### Bacterial strains and identification of biotype and serotype

We chose 271 pathogenic and 27 non-pathogenic *Y. enterocolitica *strains isolated from diarrhea patients, animals, food and the environment in China. They included 205 strains of serotype O:9, 72 of serotype O:3, 10 of serotype O:8, 5 of serotype O:5, 3 of serotype O:6, 30 and 3 of undetermined serotype (Table 1), together with 11 reference strains from Europe, the United States and Japan (Table 2). The serotypes, biotypes and pathogenesis of these strains were determined as previously described [16-18].

**Table 1:** Bio-serotypes of the 298 *Y. enterocolitica *isolates from China

Host	Pathogenic Strains						Non-pathogenic Strains						
	
	2/O:9	3/O:9	2/O:3	3/O:3	4/O:3	Total	1A/O:3	1A/O:9	1A/O:8	1A/O:5	1A/O:6,30	1A/Un*	Total
Patient	6	2	1	4	1	14				5	3		8
Swine	83	3		46		132	1	2	4				7
Dog	3			13		16						1	1
Fowl	1	1		1		3	1	1					2
Goat				2		2			2				2
Cattle		1				1		1	2				3
Fish				1		1							0
Rat	96	1		1		98						1	1
Rabbit	1					1							0
Food	1	1				2			2			1	3
Environment	1					1							0
Total	192	9	1	68	1	271	2	4	10	5	3	3	27

*: undetermined serotype

**Table 2:** Sources of the 11 reference strains

Strains No.	Bioserotype	Location	Source
52203	4/O:3	The Pasteur Institute, France	Purchased from the Pasteur Institute by the Institute of Chinese Biomedicine.
52212	4/O:9		
52211	1B/O:8		
			
Pa40134	4/O:3	Japan	Provided by Dr. H. Fukushima (Public Health Institute of Shimane Prefecture, Matsue, Japan).
ye3vp-/03	3/O:3		
ye3vp5/03	3/O:3		
ye4/03	4/O:3		
D92	2/O:5,27		
Pa12986	1B/O:8		
Ye92010	1BO:8		
			
8081	1B/O:8	Complete genome sequence of the highly pathogenic *Yersinia enterocolitica subsp. enterocolitica *8081 (Genbank: NC_008800).	

### Primer nucleotide sequences

The primers for *ail *and *foxA *were designed in our laboratory, referencing sequences from GenBank (*ail*: M29945, *foxA*: X60447), and synthesized by Shanghai Sangon Biological Engineering & Technology and Service Co., Ltd, China. The primers for *ail *amplify the entire ORF, while those for *foxA *amplify the ORF coding region from nt 28 to nt 1,461 (Table 3).

**Table 3:** Primer sequences and annealing temperatures for *ail *and *foxA*.

Target gene and primer direction		Primer Sequences (5'→ 3')	GenBank no.	Location (nt)	Amplicon length	Annealing temp.
*ail*	Forward	GGT TAT TGT ATT AGT ATT GTT	M29945	446-466	585 bp	57°C
	Reverse	CAG GTG GGT TTT CAC TAT CTG		1031-1051		
*foxA*	Forward	CTC TGC GGA AGA TAA CTA TG	X60447	389-408	1532 bp	58°C
	Reverse	ATC CGG GAA TAA ACT TGG CGT A		1899-1920		

### PCR, DNA sequencing and sequence analysis

Bacteria were cultured as previously described [18]. The bacterial DNA was extracted using a Blood & Tissue Kit (QIAGEN, USA). PCR was performed in a 200 μl volume containing 10 ng DNA template, 5U *Taq *DNA polymerase (TaKaRa, China), 0.2 mM of each dNTP, 1 μM of each forward and reverse primer, 1.5 mM MgCl_2_, 50 mM KCl, and 10 mM Tris-HCl (pH 8.3). Thermal cycling was done in a MJ PTC200 (Bio-Rad, USA) and the conditions were: one cycle of denaturation at 94°C for 5 min, followed by 25 cycles of melting at 94°C for 15 s, annealing for 30 s at various temperatures depending on the primers used (Table 3), elongation at 72°C for 30 s, and a final extension at 72°C for 10 min. Five microliters of PCR product was electrophoresed on a 1.5% agarose gel. The gel image was captured using a Gel Documentation 2000 (Bio-Rad, USA).

The specific PCR products were purified using a Gel Extraction Kit (QIAGEN, USA) and sequenced using an ABI PRISM^® ^BigDyeTM Terminator cycle sequencing Ready Reaction Kit with AmpliTag DNA Polymerase, following the manufacturer's instructions, and an ABI PRISM^® ^377XL DNA Sequencer (Applied Biosystems, Foster City, CA, USA) at TaKaRa Biotechnology (Dalian) Co., Ltd. The sequences were aligned with the reference sequences. Nucleotide sequence alignments and cluster tree construction were performed using Clustal X (Version 1.8) and MEGA (Version 4).

## Results

### General features of *ail *and *foxA*

*ail *is located on the *Y. enterocolitica *chromosome where the ORF encodes a peptide of 178 amino acids, MW: 19,548 Da [19]. There is a typical prokaryotic signal sequence at the N-terminus of the peptide [20] with a cleavage site between residues 23 and 24, where the first 23 amino acids act as a signal sequence [19].

*foxA *has an ORF of 2,129 bp encoding a protein of 710 amino acids, MW: 78,565 Da. The first 26 amino acids are a signal sequence, and a mature protein of 684 aa, MW: 75,768 Da, is formed after cleavage [14]. There is a sequence ahead of *foxA *with homology to the putative ferric ion uptake regulator (Fur) of *Yersinia *[21]. The expression of *foxA *may be regulated by iron via the Fur protein, as in other known siderophore receptors [14]. Fur may be a transcription inhibition protein acting on the ferric regulation promoter using Fe^2+^-dependent DNA binding activity homologous to that in *E. coli *[22-25].

### Analysis of *ail*

**Figure 1:**
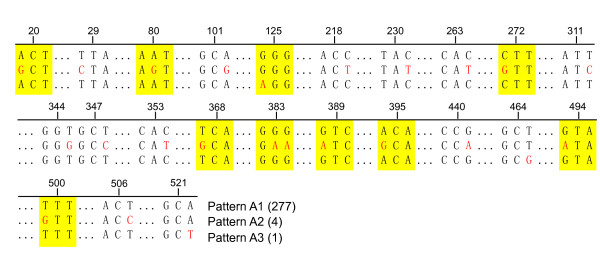
**Sequence polymorphism in *ail *from 282 isolates of pathogenic *Y. enterocolitica***. Each number on the scale indicates the site number in the ORF; red letters indicate the mutated bases; the yellow regions are missense mutations; and the other mutations are nonsense.

The entire *ail *ORF for 271 pathogenic *Y. enterocolitica *strains isolated from China and 10 reference strains were analyzed and compared to strain 8081. The data showed that all the strains can be divided into 3 sequence patterns. The Chinese isolates, 270 strains (70 of serotype O:3 and 200 of serotype O:9) and 7 reference strains (5 of O:3, one of O:9 and one of O:5,27), were sequentially identical and formed pattern A1. Four highly pathogenic strains of serotype 1B/O:8 showed identical sequences and formed pattern A2. Compared to pattern A1, pattern A2 showed 21 base mutations among which 9 were sense and 12 were nonsense mutations. In addition, one pathogenic Chinese isolate O:9 serotype (isolated from the tongue of a rat in Ningxia, 1997) showed 3 base mutations compared to the entire *ail *of pattern A1, one sense and 2 nonsense; it formed pattern A3 (Fig. 1). This new *ail *genotype was submitted to Genbank and given the accession number GU722202.

### Analysis of *foxA*

**Figure 2:**
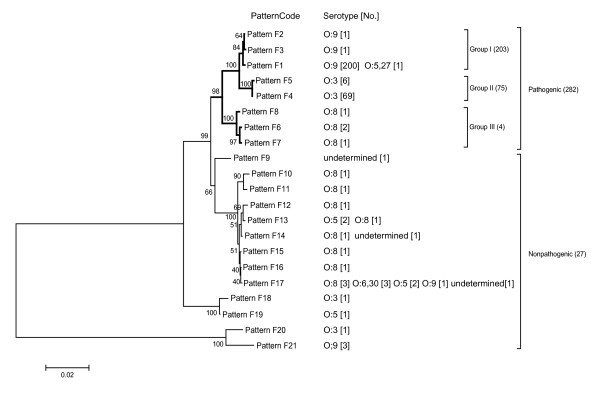
**Phylogenetic tree of *foxA *from 309 isolates of *Y. enterocolitica***. Among the 309 isolates studied, 282 were pathogenic and the others were nonpathogenic. [No.]: the number of the strains of the same serotype in the pattern.

**Figure 3:**
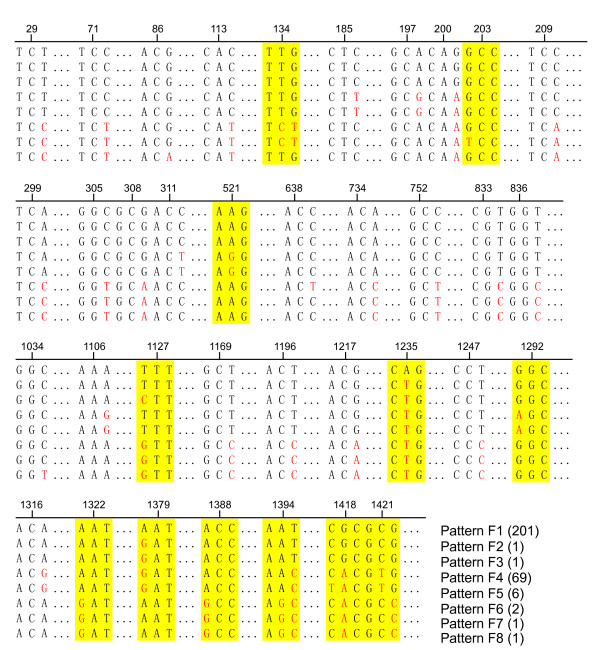
**Sequence polymorphism in *foxA *from 282 isolates of pathogenic *Y. enterocolitica***. The numbers on the scale indicates the site numbers in the ORF; red letters indicate mutated bases; the yellow regions are missense mutations; and the other mutations are nonsense.

Analysis of the primary coding region of *foxA *from nt 28 to nt 1,461 in 271 pathogenic *Y. enterocolitica *strains isolated from China and 11 reference strains showed that all the strains can be divided into 3 groups including 8 sequence patterns (Fig. 2). Group I comprised patterns F1, F2 and F3 and included 201 serotype O:9 strains isolated from China and 2 reference strains (one strain O:9 and one O:5,27). Among these, 199 strains of O:9 and 2 reference strains showing identical sequences formed pattern F1, and 2 other strains of O:9 with 2 and 3 base mutations formed patterns F2 and F3. Group II comprised patterns F4 and F5, and included 70 Chinese isolates and 5 reference strains of serotype O:3. Sixty-nine serotype O:3 strains (67 Chinese isolates and2 reference strains) showing identical sequences formed pattern F4; and 6 other strains of O:3 had one base mutation and formed pattern F5. Group III comprised five reference strains including patterns F6, F7 and F8. Pattern F6 (2 Japanese strains) had 2 base mutations compared to pattern F7 (52211). Compared to pattern F7, pattern F8 (8081) had 5 base mutations (Fig. 3).

To analyze *foxA *polymorphism in *Y. enterocolitica *overall, we chose 27 strains of non-pathogenic *Y. enterocolitica *as controls (Table 1). The results showed 13 sequence patterns for the 27 strains with 10's to 100's more polymorphic sites and no apparent regularity. This indicated that *foxA *was less polymorphic and more conserved in pathogenic strains than in non-pathogenic strains.

## Discussion

Only pathogenic *Y. enterocolitica *contains *ail*, which confers a bacterial invasion and serum resistance phenotype, that is an important virulence marker on the chromosome [6,19]. The entire ORF of *ail *was sequenced and analyzed from strains from different sources and biotypes and serotypes. The data showed that the 282 pathogenic *Y. enterocolitica *formed 3 sequence patterns (Fig. 1); the strains were pathogenic O:3 and O:9 isolated from various hosts in China and the reference strains. Only one Chinese isolate formed pattern A3, a new *ail *genotype submitted to Genbank and given the GenBank accession number GU722202. When it was compared to the sequence of pattern A1, three base mutations were found, one sense and two nonsense. We presume that pathogenic *Y. enterocolitica *had 2 original *ail *patterns, A1 represented in serotypes O:3 and O:9 and A2 represented in bio-serotype 1B/O:8; pattern A3 may be a mutation of A1. Pathogenic *Y. enterocolitica *can be divided into a high-pathogenicity group (*Y. enterocolitica *biogroup 1B) and a low-pathogenicity group (*Y. enterocolitica *biogroups 2 to 5) on the basis of the lethal infectious dose in the mouse model [26]. The typing of *ail *in this study is consistent with this grouping of pathogenic strains. Therefore, we presumed that lethality in mice (50% lethal dose <1,000 microorganisms) not only depends on the presence of the yersiniabactin (*ybt*) locus, which carries the genes for biosynthesis, transport and regulation of the siderophore yersiniabactin [8,27-29], but is also closely related to the *ail *sequence. We conjecture that synergism with *ail *is necessary for *Y. enterocolitica *pathogenesis.

*ail *is not only an important virulence gene for pathogenic *Y. enterocolitica*, but also harbors highly conserved sequences, mutation of which may change the virulence of the bacterium. For instance, in the 1B/O:8 strain, which is highly lethal to mice, the *ail *belongs to pattern A2, while *ail *in other pathogenic bioserotype strains belongs to pattern A1. So we believe that a change in *ail *is closely related to the pathogenesis of the strain. A pathogenic O:9 strain isolated from *Cricetulus triton *in Ningxia contains *ail *pattern A3, the sequence of which has 3 site mutations, only one being a sense mutation. Further study is needed to see whether amino acid change alters the function of Ail protein or bacterial virulence.

Analysis of the 1,434 base pairs of the *foxA *primary coding region showed that the *foxA *sequence correlated with the biotype and serotype of pathogenic *Y. enterocolitica*. Comparing the primary sequences of groups I and II, 13 base mutations at fixed positions were found; 5 were sense and 8 were nonsense mutations, indicating that the primary difference in the pathogenic *Y. enterocolitica foxA *was located in these 13 sites. Strain 8081 showed 26 base mutations compared to F1 and 31 compared to F4. From these findings we presume that pathogenic O:3 and O:9 have similar *foxA *sequences (Fig. 2) and mutation sites additional to strain 8081 bio-serotype 1B/O:8 (Fig. 3). Thus, there is a correlation between pathogenesis and the different bio-serotypes of *Y. enterocolitica*. More mutation sites and no obvious regulation were found in non-pathogenic *Y. enterocolitica foxA*, although some strains showed an identical *foxA *sequence type (Fig. 2). The identical sequence patterns of the pathogenic *Y. enterocolitica *strains isolated from different areas, at different times and from different host sources show the *foxA *sequence pattern to be closely correlated to pathogenesis. Unfortunately, fewer strains from outside China were used, which is a limitation of the study and needs adding strains for future study.

*ail *is a primary marker for pathogenic *Y. enterocolitica *and is an important tool for detecting it, making it a very important gene to analyze. Some scholars have established a real-time PCR assay to detect *Y. enterocolitica *using *ail *or *ystA *as the target gene [30-33]. According to the current identification standards, strains having no *ail *and harboring *ystB *isolated from diarrhea patients are classified as non-pathogenic. However, other researchers believe that strains harboring *ystB *are pathogenic and cause the diarrhea, as inferred from epidemiology and the etiology of disease outbreaks [34,35]. Therefore, it is necessary to detect other conserved genes as a complement to these markers because reliance on only *ail *and *ystA *to detect pathogenesis remains questionable. We chose to detect *foxA*, which is found in both pathogenic and non-pathogenic *Y. enterocolitica*. The results showed that both *ail *and *foxA *were conserved together in pathogenic strains and can therefore be used to confirm the detection of pathogenic *Y. enterocolitica*. Currently, we are attempting to extract bacterial DNA from clinical specimens to detect *foxA *in order to identify *Y. enterocolitica *directly from humans and other animals; and we have some preliminary data (unpublished).

Almost all *Y. enterocolitica *carry *foxA *while pathogenic strains carry *ail*. It is very important for real-time PCR detection of *Y. enterocolitica *to study sequence polymorphism in *ail *and *foxA*. It will be helpful to design specific primers and probes in the conserved region in order to develop real-time or traditional PCR methods. We are trying to establish a duplex real-time PCR to detect *Y. enterocolitica *from clinical samples and to confirm its pathogenicity. Designing specific primers for *foxA *and *ail *in a combined detection system is valuable for increasing sensitivity and specificity in the detection of pathogenic *Y. enterocolitica*.

## Conclusion

Analysis of polymorphisms in *ail *and *foxA *of pathogenic *Y. enterocolitica *strains from different times and regions showed *ail *to be an important virulence gene for pathogenic *Y. enterocolitica*, and that it has a highly conserved sequence. The gene encoding the ferrioxamine receptor, *foxA*, is also conserved in pathogenic strains, where 2 primary sequence patterns were found. More strains from outside China are needed for further study.

## Authors' contributions

YH did most of the PCR work and DNA sequencing. XW analyzed the sequences. ZC did the data clustering and construction of phylogenetic trees. YY and YX identified the biotypes and serotypes of strains. LT wrote the paper. BK and XJ participated in discussion of the study. HJ designed and coordinated the study and drafted the manuscript. All the authors read and approved the final manuscript.
